# Invasive Australian *Acacia* seed banks: Size and relationship with stem diameter in the presence of gall-forming biological control agents

**DOI:** 10.1371/journal.pone.0181763

**Published:** 2017-08-16

**Authors:** Matthys Strydom, Ruan Veldtman, Mzabalazo Z. Ngwenya, Karen J. Esler

**Affiliations:** 1 Department of Conservation Ecology and Entomology, Stellenbosch University, Matieland, South Africa; 2 Centre of Excellence for Invasion Biology, Stellenbosch University, Matieland, South Africa; 3 South African National Biodiversity Institute, Kirstenbosch National Botanical Garden, Cape Town, South Africa; 4 Biometry, Agricultural Research Council, Stellenbosch, South Africa; Brigham Young University, UNITED STATES

## Abstract

Australian *Acacia* are invasive in many parts of the world. Despite significant mechanical and biological efforts to control their invasion and spread, soil-stored seed banks prevent their effective and sustained removal. In response South Africa has had a strong focus on employing seed reducing biological control agents to deal with Australian *Acacia* invasion, a programme that is considered as being successful. To provide a predictive understanding for their management, seed banks of four invasive Australian acacia species (*Acacia longifolia*, *A*. *mearnsii*, *A*. *pycnantha* and *A*. *saligna*) were studied in the Western Cape of South Africa. Across six to seven sites for each species, seed bank sizes were estimated from dense, monospecific stands by collecting 30 litter and soil samples. Average estimated seed bank size was large (1017 to 17261 seed m^-2^) as was annual input into the seed bank, suggesting that these seed banks are not residual but are replenished in size annually. A clear relationship between seed bank size and stem diameter was established indicating that mechanical clearing should be conducted shortly after fire-stimulated recruitment events or within old populations when seed banks are small. In dense, monospecific stands seed-feeding biological control agents are not effective in reducing seed bank size.

## Introduction

Australian *Acacia* have become naturalised and invasive in many parts of the world [1]. In their invaded ranges, they change the structure and functioning of ecosystems, decreasing species richness and abundance [2] and impacting ecosystem services [3]. Especially in South Africa these invasive alien plants have impacted a vast area of natural and agricultural land [4]. This has consequently resulted in significant management effort in an attempt to control the invasion [5]. A particular challenge to control efforts of invasive Australian *Acacia* is their ability to accumulate persistent seed banks [6–8]. The importance of managing seed banks of invasive Australian *Acacia* has also been recognised in Australia, Israel and Portugal [9–11].

The persistence of Australian *Acacia* seeds is due to a water impermeable testa which physically imposes seed dormancy [12] and is broken only when the seed coat is adequately damaged, generally through a heat pulse (e.g. fire) [6]. The degree of initial seed dormancy (ranging between 0 to 100%) is determined by relative humidity within the seed pods during seed maturation [13]. Consequently, a whole seed crop may be lost to germination, or decay within the first year after production, or remain dormant in the soil. Seed survival probability increases with time in the soil [14]; some seeds can persist for 50 or more years [6]. Seed banks thus allow invasive Australian *Acacia* to survive disturbances (such as fire) in time and space and to re-establish in environments temporarily free from management [7,8].

Management efforts in South Africa have included mechanical, chemical and biological control [5]. The biological control agents released on invasive Australian *Acacia* include five *Melanterius* spp. (seed feeding weevils), two *Trichilogaster* spp. (bud galling wasps), two *Dasineura* spp. (flower galling flies) and one *Uromycladium* spp. (gall rust fungus) [15]. The impact of these biological control agents on seed production is described as extensive (almost no seeds surviving) or considerable (>50% seed reduction) [15]. Consequently, biological control programs have been assessed as successful, and it is maintained that biological control is the only sustainable and cost effective method for controlling the Australian *Acacia* invasion [5,16].

Despite biological and mechanical control efforts, the ability of Australian *Acacia* to accumulate persistent seed banks remains a challenge to their effective removal and sustained absence [8,17]. Recruitment from seed banks results in the need for repeated follow-up clearing operations [7]. This leads to decreased clearing programme effectiveness and drastically increases already expensive clearing costs [18].

Australian *Acacia* can be divided into ant-dispersed and bird-dispersed species [17,19]. Ant-dispersed species have a persistent seed bank with a clear germination response to fire, while bird-dispersed species have a seed bank intermediate between a transient and persistent type without a definite fire germination response [17]. In the absence of fire, ant-dispersed seed display higher levels of dormancy and lower levels of germination and decay when compared to bird-dispersed species [20]. Consequently ant-dispersed species tend to accumulate larger seed banks with a greater proportion persisting after aboveground clearance [7]. *Acacia longifolia*, *A*. *mearnsii*, *A*. *pycnantha* and *A*. *saligna* all have ant-dispersed seeds, while *A*. *cyclops* and *A*. *melanoxylon* are bird-dispersed [17].

Richardson and Kluge [17] have stressed the importance of estimating Australian *Acacia* seed bank size, as well as determining how seed banks relate to stand characteristics (e.g. stand age and density), for management. Seed bank data combined with stand characteristic data are also required to make sense of the estimated damage levels of biological control agents. However, little or no data on the seed banks of many of the Australian *Acacia* before and after the release of their respective biological control agents are available [17, 21]. This is not unique to Australian *Acacia*, as the seed banks of invasive species globally are generally poorly understood [22].

We investigated the seed banks of the most problematic ant-dispersed Australian *Acacia* (*A*. *longifolia*, *A*. *mearnsii*, *A*. *pycnantha* and *A*. *saligna*), in the Western Cape of South Africa [4]. These species were chosen as they potentially pose the largest obstacle in terms of their seed banks to managers. The size of the seed banks of these species was determined at a regional scale (Western Cape of South Africa) and seed bank size was related to stand characteristics (including the presence of released gall-forming agents). A regional scale was chosen as this is the relevant scale at which management decisions are made, and it is the average response of seed banks in the presence of biological control agents that will determine management action. The following questions were asked:

1. What is the size of the seed bank of ant-dispersed Australian *Acacia* in the presence of gall-forming biological control agents?
2. What is the relationship between the seed bank, tree density and stem diameter of ant-dispersed Australian *Acacia*?

## Methods

### Study sites

For each *Acacia* species (*A*. *longifolia*, *A*. *mearnsii*, *A*. *pycnantha* and *A*. *saligna*), six to seven sites were selected for study in the Western Cape of South Africa (S1 Fig). A site was defined as a monospecific stand of approximately one hectare or larger with near complete or complete canopy cover (75 to 100%). Despite this canopy cover, the selected sites varied in tree density and stem diameter. Monospecific stands were chosen to reduce the number of potential factors that can influence the seed bank (such as interactions with other plant species). All sites were located on privately owned land and permission was acquired from all landowners to conduct research on their premises. Furthermore, the studied species are invasive in South Africa and are not protected or endangered. Mean annual rainfall over the sampled range varied between 412 to 955 mm with most of the rain falling in the winter months (57 to 80%) (S1 Table). Mean annual temperature over the sampled range was recorded as 15 to 19°C with the average minimum temperature of the coldest month recorded as 5 to 10°C and average maximum temperature of the warmest month recorded as 27 to 33°C (S1 Table).

### Seed bank sampling

Seed bank sampling was conducted during July 2013. Thirty litter and soil samples were taken at each site according to the random sampling technique described in Strydom et al. [8]. A ring with a diameter of 6 cm was placed on the litter surface and all unconsolidated litter material inside the ring was collected. Afterwards a soil sample was taken at the same point with a soil corer (with a diameter of 5 cm and a length of 15 cm). Soil samples were taken to a depth of 15 cm as the largest portion of the seed bank (>90%) of Australian *Acacia* is situated in the top 10 cm of the soil profile [8]. Samples were sieved through a 2 mm mesh and all the *Acacia* seeds were collected and counted. The average litter, soil, and total (litter + soil) seed bank size for the Western Cape was calculated.

### Stem diameter and tree density

At each sampling point, the relationship between tree density and the seed bank was determined by counting the number of trees within a circle (1.5 m radius), with the seed bank sample taken as the midpoint. Trees with stems located outside the circle but whose canopies intersected the seed bank sampling point were also counted. The largest proportion of Australian *Acacia* seeds fall directly beneath their canopy [6,11]. Therefore, the relationship between tree size and the seed bank was assessed through measuring (within the same circle in which tree density was assessed) the diameter at breast height (DBH) of trees whose canopies intersected the seed bank sampling point. The DBH of trees with stems located outside the circle but whose canopies intersected the seed bank sampling point were also measured. If the stem diameter of all the trees in the circle was not already measured, the DBH of up to three additional trees within the circle was measured. This was done to take into account the effect of germination on seed bank size. From these data, average stem diameter at each sample location was calculated. Since stem diameter data also serve as a proxy of tree age [23], it was used to assess the development of seed banks over time. To do this, stem diameter of each species was divided into different size classes and average seed bank size for each class calculated.

### Seed viability

To determine seed viability at each sample site, seeds from all 30 soil samples were used. Depending on availability, 100 seeds were randomly chosen from all the seeds extracted from the soil samples and then surface-sterilised by washing them in a 3.5% sodium hypochlorite solution [24]. Seed coats were then chipped at the distal end and 4 petri-dishes (each containing two filter paper discs moistened with 10 ml water) each were allocated 25 of these seeds [25]. Petri-dishes were placed in plastic bags to prevent moisture loss [25] and incubated at 25°C in the dark [12]. After three days, seeds were checked for germination and thereafter daily for two weeks. Germination and thus seed viability was assumed if the radicle was at least 1 mm long [24]. These data were used to calculate seed viability for each *Acacia* species studied.

### Biological control

The gall-forming biological control agents of *A*. *saligna*, *A*. *pycnantha* and *A*. *longifolia* have established over these invasive alien plants entire distribution range within South Africa [15,26,27]. For each of these agents enough time has passed (since their declared establishment throughout their hosts’ distribution range) for them to have reach densities to have visual and measured impact on their hosts populations (e.g. reduced canopy cover and seed production) within South Africa [28–32]. The assessment of the average seed bank size of invasive Australian *Acacia* at different size classes and therefore at different time intervals (i.e. creating a chronosequence for the seed banks of the investigated species), represents the trends of these plants’ seed banks over time under the average impact of their gall-forming biological control agents. Consequently, this will be a measurement of the average behaviour of the seed bank of these invasive Australian *Acacia* under the normal dynamics of these plants with their associated agents within the Western Cape. The average time of infection after disturbance events (e.g. fire) as well as average accumulation time of these agents is therefore also taken into account.

The gall-forming agent of *A*. *mearnsii* has been present in South Africa for a short period and has not spread throughout the plants distribution range [15]. However, the seed bank data of *A*. *mearnsii* has been collected to serve as baseline data for future comparisons. The development of invasive Australian *Acacia* seed banks in the presence of gall-forming agents that can impact on their seed production is therefore specifically investigated for *A*. *longifolia*, *A*. *pycnantha* and *A*. *saligna*.

Previously published seed bank size estimates before and after the release of the respective gall-forming biological control agents were collated for each of the studied species (Table 1 and S2 Table). Seed bank estimates of previously published data were only included if they were based on samples of 30 or more, as this is the minimum sample size to accurately estimate seed bank size [8]. Comparisons between current and previous data were made only if data from at least three different localities were available in the pre- and post biological control categories. This was done to assess whether the released gall-forming biological control agents have had a measurable impact on the seed bank capacity of their Australian *Acacia* hosts.

**Table 1:** Seed bank size of Australian *Acacia* before and after the release of their gall-forming biological control agents.

Species	Biological control agent	Year of agent release *	Seed bank	
			Before release	After release
*Acacia longifolia*	*Trichilogaster acaciaelongifoliae*	1982	2110^(^^3^^)^	2078–2901^(^^4^^)^
*Acacia mearnsii*	*Dasineura rubiformis*	2006	-	-
*Acacia pycnantha*	*Trichilogaster signiventris*	1987	-	-
*Acacia saligna*	*Uromycladium tepperianum*	1987	145–45800^(^^1^^,^^3^^)^	137–46355^(^^2^^,^^5^^,^^6^^)^

Indicates the gall-forming biological control agents released on *Acacia longifolia*, *A*. *mearnsii*, *A*. *pycnantha* and *A*. *saligna*. The year of first release of the different biological control agents in South Africa is shown. Seed bank size (seeds m^-2^) of the different Australian *Acacia* recorded before and after the release of their respective biological control agents is also indicated.

^1:^ Holmes et al. 1987;

^2:^ Jasson 2005;

^3:^ Milton & Hall 1981;

^4:^ Pieterse & Cairns 1986,

^5:^ Strydom 2012,

^6:^ Strydom et al. 2012.

* Data from Impson et al. 2011.

Seed bank estimates of Morris [30] sampled during 1991 were also included in the before *U*. *tepperianum* release category. The gall rust fungus was released during 1989 at these sites but only became abundant at these sites during 1993 / 1994 and did not show a visible impact until 1995 / 1996 [30]. Subsequent seed bank estimates from 1992 to 1995 of Morris [30] were not included, as the seed banks were repeatedly sampled at the same points along permanent transects. Consequently, the data from these estimates (1992 to 1995) could show a decline in seed bank size simply because the same seed stock was depleted over time.

### Statistical analysis

A generalised linear mixed model (GLMM) was fitted to the seed bank data to determine whether tree density and stem diameter significantly affect seed bank size. Quantile regressions were then used to establish the relationship between significant variables with the seed bank over the environmental gradient. A Wilcoxon rank sum test was conducted to determine whether seed banks of the studied Australian *Acacia* differed before and after the release of their gall-forming biological control agents. All statistical analyses were conducted in R 3.1.2 [33].

#### Generalised linear mixed models

To determine the response of the seed bank to tree density and stem diameter, generalised linear mixed models (GLMMs) were used with count data as the response variable and site as a random effect [34]. Initially, models with a log link function and a Poisson error distribution were fitted to the data. However, the data showed more variation than what would be expected for a Poisson distribution hence a negative binomial error distribution was used in all models to account for overdispersion [35]. The negative binomial error distribution is widely used in GLMMs to model data with overdispersion [36]. All models were fitted using the lme4 package [37]. Fixed effects were standardized to remove the effect of scale on parameter estimates [35] and maximum likelihood estimation was used to obtain model parameters [38].

The maximal model contained tree density, stem diameter and species as explanatory variables and site as a random effect. A submodel set was generated from the maximal model from which a 95% model confidence set was obtained [35] via dredging, an automated process executed by the R package MuMIn [39]. Models were compared through the use of information theoretic (I-T) model procedures based on Akaike’s information criterion (AIC) [40]. The model second order AIC value, the AICc, was used to select the best model; i.e. the model with the smallest information loss was chosen as the best model. If the best model had a corrected Akaike weight (AICcWt) of less than 0.9, model averaging was conducted on the 95% model confidence set to obtain parameter estimates, parameter confidence intervals and relative parameter importance [35].

#### Quantile regression

The set of five hierarchical models proposed by Huisman et al. [41] was used to determine the response curve of the seed bank (litter + soil) to stem diameter over the sampled environmental gradient for each studied species. These models were used as they have been shown to be appropriate to detect non-linear response curves along environmental gradients [42]. The most complex model, from which four simpler models can be derived, is defined as follows by Huisman et al. [41]:

$$\begin{array}{ccc} \begin{array}{ccc} y & = & M \end{array} & \frac{1}{\begin{array}{ccc} 1 & + & e^{a+bx} \end{array}} & \frac{1}{\begin{array}{ccc} 1 & + & e^{c-dx} \end{array}} \end{array}$$

where y and x are the response and explanatory variables respectively, *a*, *b*, *c* and *d* are the parameters to be estimated and *M* which is a constant refers to the maximal attainable value for y. The value of *M* was set by determining the highest recorded number of seeds in the seed bank of each species. Quantile regression models at the 95^th^ percentile were fitted to the data using all five models. The best fitting model was then determined via the AIC wherein the best model is that with the smallest value. This was done via the R package quantreg [43]. Regression quantiles at 95^th^ percentile were used as this method reduces the impact of multiple unmeasured factors on the regression analysis [44]. Therefore, using regression quantiles at the 95^th^ percentile will allow inferences to be made of the relationship between two variables in the presence of high environmental variation [44]. Increasing the percentile used also gives a measure of control over the impact of unmeasured variables (i.e. the higher the percentile the greater the correction for the effect of the variable) [44]. Use of the 95^th^ rather than the 99^th^ percentile is preferred in order to decrease the potential impact of outliers [45].

The extreme seed bank values (>6000 seeds m^-2^) in the *A*. *longifolia* data set were removed, because there were too few data points in the tails of the data set to compensate for the effect of the extreme values. However, the analyses with these sites are included in the supplementary material (S1 File and S2 Fig).

#### Wilcoxon rank sum test

To determine whether seed banks of the studied species differed before and after the release of their respective gall-forming biological control agents, a Wilcoxon rank sum test was conducted. This non-parametric test was used as the data were not normally distributed.

## Results

### The seeds in the litter, soil and seed bank

**Fig 1:**
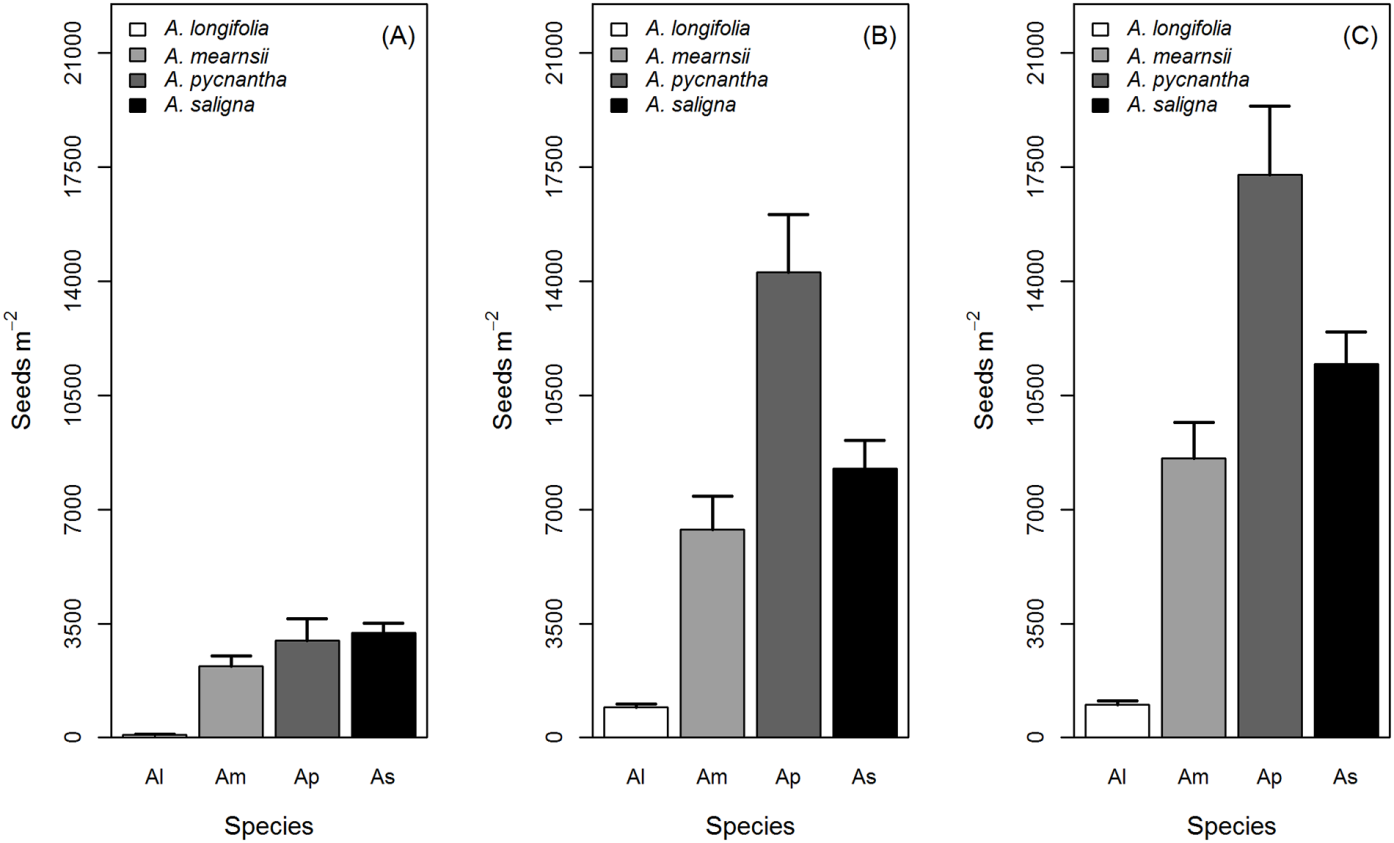
Seeds in the litter, soil and seed bank indicates the seeds surviving the pre- and post dispersal phases. Average seeds m^-2^ (+SE) in the litter (A), soil (B) and seed bank (litter + soil, C) of 4 ant-dispersed Australian *Acacia* in the Western Cape of South Africa.

A range of seed numbers was present in the litter, soil and seed bank (i.e. litter + soil) of all the studied species (Fig 1). In the litter, *A*. *saligna* had the most seeds on average (3213 seed m^-2^), followed by *A*. *pycnantha* (2987 seed m^-2^), *A*. *mearnsii* (2187 seed m^-2^) and lastly *A*. *longifolia* (91 seed m^-2^). In the soil, *A*. *pycnantha* had on average the most seeds (14274 seed m^-2^), followed by *A*. *saligna* (8248 seed m^-2^), *A*. *mearnsii* (6378 seed m^-2^) and *A*. *longifolia* (926 seed m^-2^). Overall (soil and litter samples combined), *A*. *longifolia* had on average the smallest seed bank (1017 seeds m^-2^) with *A*. *pycnantha* having the largest (17261 seeds m^-2^), followed by *A*. *saligna* (11460 seeds m^-2^) and *A*. *mearnsii* (8564 seeds m^-2^).

### Seed bank relationships with tree density, stem diameter and species identity

Species identity had a significant effect on seed bank size (Table 2). The evidence that there is a significant difference between the seed banks of the different species is supported by the confidence interval of their parameter estimates not containing zero. Furthermore, the sum of the Akaike weights for the models in which species was present as a fixed effect is 1 (Table 3), further indicating a significant effect of species identity on seed bank size.

**Table 2:** Analyses relating seed bank size to stem diameter and tree density.

Parameter	Estimate	Standard error	p -value	Confidence interval	RI
Intercept	6.48	0.37	< 0.001	(5.76, 7.21)	-
Stem diameter	0.14	0.06	0.019	(0.02, 0.26)	1
Tree density	0.01	0.07	0.938	(-0.13, 0.14)	0.27
*Acacia mearnsii*	1.87	0.54	< 0.001	(0.80, 2.94)	1
*Acacia pycnantha*	2.50	0.55	< 0.001	(1.44, 3.58)	1
*Acacia saligna*	2.72	0.55	< 0.001	(1.65, 3.80)	1

Results of model averaging over the fitted models with the seed bank as response variable and site as random effect. The parameter estimates, unconditional standard errors, parameter significance, parameter estimate confidence intervals and relative importance (RI) of the explanatory variables after model averaging are shown.

**Table 3:** Candidate models over which model averaging was conducted.

Model	*k*	AICc	Δ_i_	*ω*_i_	Acc *ω*_i_	LL
Stem diameter + species	7	13587.68	0.00	0.73	0.73	-6786.76
Stem diameter + tree density + species	8	13589.71	2.04	0.27	1	-6786.76

Best candidate models (95% confidence set) predicting seed bank size. Model AICc values, model weights (*ω*_i_), cumulative model weights (acc *ω*_i_) and Laplace approximations (LL) is shown. Model averaging was conducted over the candidate models and the results are shown in Table 2.

Stem diameter and tree density were positively related to seed bank size, but with only stem diameter being significantly so (Table 2). The lack of evidence that tree density affects seed bank size is further supported by the confidence interval of its parameter estimate containing zero, including a relative importance of 27% to stem diameter and species identity (Table 2). There is strong evidence for the effect of stem diameter on the seed bank as the confidence interval of its parameter estimate did not contain zero. The effect of stem diameter on the seed bank is further supported as it has a relative importance of 1 (Table 2).

**Fig 2:**
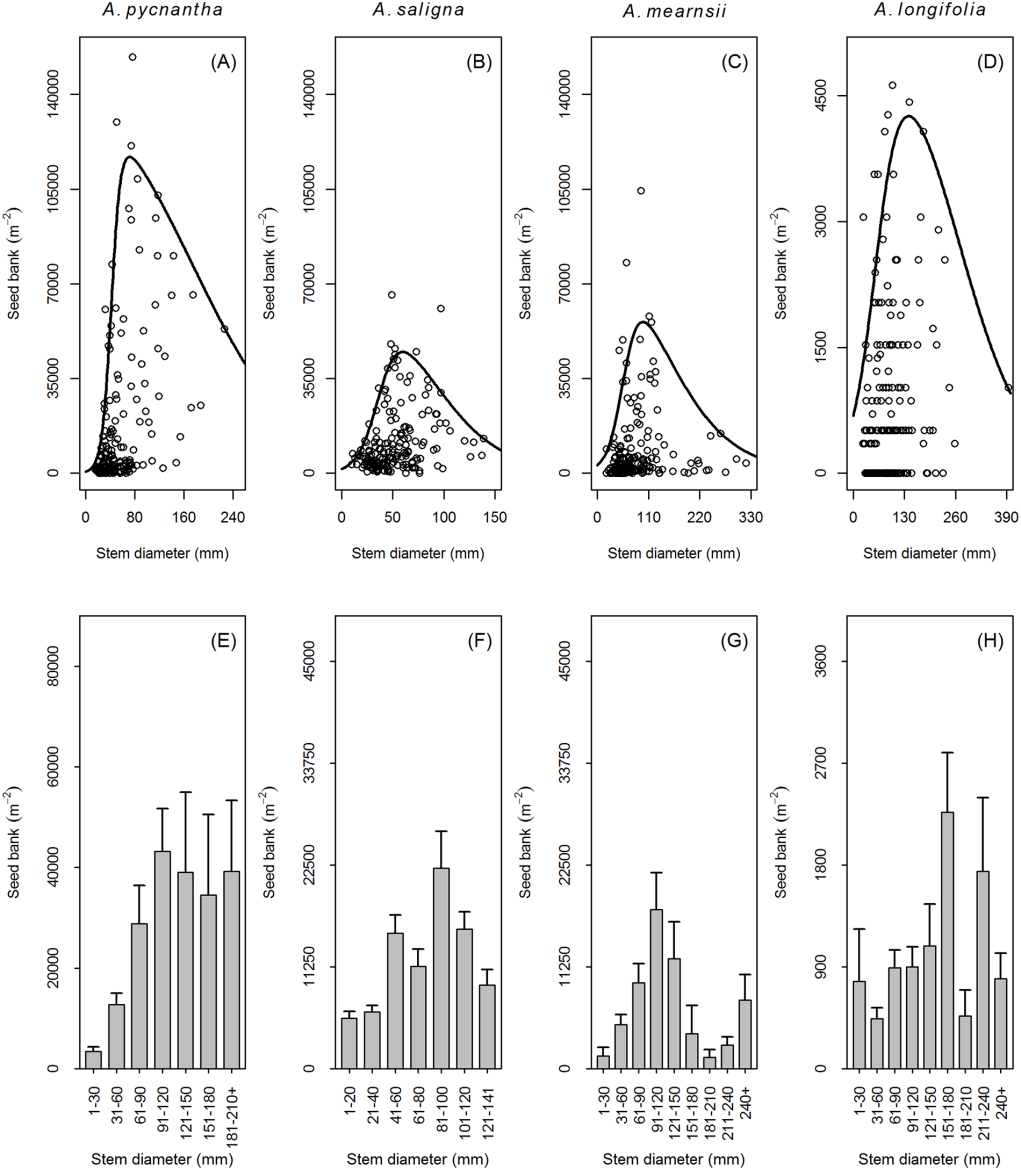
The relationship between seed bank size and stem diameter indicates when management should be conducted. (A-D) The relationship between the seed bank (m^-2^) and stem diameter of four ant-dispersed Australian *Acacia* (*Acacia pycnantha*, *A*. *saligna*, *A*. *mearnsii*, *A*. *longifolia*). Relationships were estimated by quantile regression at the 95^th^ percentile. (E-H) The average seed bank size (m^-2^) (+SE) for stem diameter classes of the studied species.

The seed banks of all the studied species showed an initial increase with an increase in stem diameter until a maximal point was reached after which seed bank size decreased with a further increase in stem diameter (Fig 2A–2D). Average seed bank size of stem diameter classes of all the studied species generally followed the same pattern of increase and decline with an increase in stem diameter class size as described above for their associated quantile regressions (Fig 2E–2H). *Acacia pycnantha* had the greatest potential seed bank capacity (indicated by the regression line) overall with average stem diameter as the explanatory variable followed by *A*. *saligna*, *A*. *mearnsii* and lastly *A*. *longifolia* (Fig 2A–2D). Potential seed bank size of *A*. *pycnantha*, *A*. *saligna*, *A*. *mearnsii* and *A*. *longifolia* is at a maximum when stem diameter for these species are 72 mm, 60 mm, 98 mm and 140 mm respectively (Fig 2A–2D). At the maximum point with stem diameter as the explanatory variable, the seed bank of *A*. *pycnantha*, *A*. *saligna*, *A*. *mearnsii* and *A*. *longifolia* is estimated to be 116895 seeds m^-2^, 44782 seeds m^-2^, 55821 seeds m^-2^ and 4256 seeds m^-2^.

### Seed viability

Seed viability of all the studied species was high. The average seed viability of *A*. *longifolia*, *A*. *mearnsii*, *A*. *pycnantha* and *A*. *saligna* was 96%, 98%, 94% and 95% respectively.

### Biological control

The galling agents of *A*. *longifolia*, *A*. *saligna* and *A*. *pycnantha* were present at all of their respective studied sites, while that of *A*. *mearnsii* was only present at two (Rivendale and Avondvrede) of the six studied sites.

No trend of decline was apparent when comparing the current study’s seed bank estimates of *A*. *saligna* (4144 to 28877 seeds m^-2^) with seed bank size estimates of previously published data either before (145 to 45800 seeds m^-2^) or after (137 to 46355 seeds m^-2^) the release of its agents (Table 1). A Wilcoxon sum rank test indicated that seed bank scores before *U*. *tepperianum* was released were not statistically different to the seed bank scores of the current study after *U*. *tepperianum* was released (*W* = 34, p = 0.89). Comparisons for the other species could not be made either because of a complete lack of data or the collected data did not meet the requirements as stated in the methods section (Table 1 and S2 Table).

## Discussion

Seed banks of invasive, ant-dispersed Australian *Acacia* (hereafter Australian *Acacia*) in South Africa are still accumulating over time in dense monospecific stands, despite the presence of gall-forming biological control agents which have been present in South Africa for 7 to 31 years [15], depending on the species. Where adequate assessments of seed banks of *A*. *saligna* were made prior to the introduction of its gall-forming biological control agent, no significant decline in seed bank size capacity was observed. Seed bank size of Australian *Acacia* initially increased with stem diameter until a stem diameter was reached after which seed bank size started to decline. Australian *Acacia* populations should be cleared shortly after disturbance events to prevent the accumulation of large persisting seed banks.

Seed bank sizes of all studied species were generally estimated to be large (>1000 seeds m^-2^) and viable (>94%). Australian *Acacia* seed banks of similar size have been shown to recruit seedlings at densities of 19 to 1200 seedlings m^-2^ [6,7,24,46,47,48] of which only 10 seedlings m^-2^ are required to form a closed canopy [49]. Therefore, enough seeds are located in the seed banks of these species to replace existing stands. Furthermore, many seeds were sampled in the litter on top of the soil surface, indicating recent addition of seeds to the seed bank that have survived the pre- and post-dispersal phases during recent seed production seasons. Therefore, despite some loss of seeds to gall-forming biological control agents [15], plus other biotic and abiotic factors such as predation by rodents and dispersal by wind [6], many seeds are still being incorporated into the seed bank of all studied species.

The clear relationship established between seed bank size and stem diameter of the studied Australian *Acacia* further supports the observation that many seeds are currently added to the seed bank. Stem diameter is a proxy for stand age [23] and therefore its relationship with seed bank size shows how seeds accumulate over time, as the stands mature, to a maximal point. The decline in the seed bank size after a maximal point is reached indicates that seed loss from the seed bank is high and constant over time. Beyond the maximal point seed input is less than seed loss, and therefore seed banks decrease in size, while the increase in seed bank size before this maximal point shows that annual seed input into the seed bank is high. This trend indicates that most seeds remain within the seed bank for only a short period of time. The short residence time can be ascribed to seeds being lost to either germination or decay [6,14]. Consequently, only a small proportion of the produced seeds remaining dormant for 50 or more years [6]. Furthermore, many seeds are still produced and survive over time, therefore seed banks are not residual. In other words, seed banks are not the legacy of seed input before their biological control agents were released, but are the consequence of current annual seed input in the presence of these agents.

The increases in seed bank size with an increase in stem diameter can be explained by the increased reproductive capacity of Australian *Acacia* trees as they mature [6,50,51]. However, overall seed input will eventually start declining as trees start producing less seeds due to senescence [6]. Therefore, the difference in seed bank capacity of the studied Australian *Acacia* species can be attributed to the above mentioned factors differing between them [6,11,14,52,53].

The model for development of the seed bank over time for the invasive Australian *Acacia* investigated here is similar to that established by Auld [54] for the seed bank of *A*. *suaveolens* in its native environment in Australia using a seed bank dynamics model. His model shows that seed bank size and the long-term survival of seeds in the soil (in the absence of fire) are largely dependent on initial fruiting events. Auld [54] indicated that the size of the seed bank of *A*. *suaveolens* is dependent on initial population size with either a increase or decrease in population size leading to a corresponding increase or decline in seed bank size. This suggests that seed banks of invasive Australian *Acacia* in the Western Cape of South Africa are larger and have greater longevity compared to those in their native ranges because of larger initial population sizes. Larger seed bank sizes of invasive Australian *Acacia* can therefore be explained by competitive release from other competitive plants rather than natural enemy release *per se*. The superior competitive ability of invasive Australian *Acacia* over native fynbos plant species after fire is well documented in South Africa, Portugal and Chile [2].

Based on this explained development of the seed bank over time, seed reducing biological control agents will have to significantly decrease seed production during the initial post-fire fruiting event, when the seed bank will be actively accumulating, to effectively reduce seed bank size and the long-term survival of seeds in the soil. However, the abundance of seed-reducing biological control agents is greatly reduced or eliminated during a fire [15,51,55] as well as after mechanical clearing events [56]. Consequently, these agents will have to re-establish within regenerating Australian *Acacia* populations. This process of population regeneration of the seed-reducing biological control agents takes several years [28–30]. Australian *Acacia* are, however, able to reproduce within two years with large seed crops being produced after five [6]. Therefore, the released biological control agents are either absent or at very low numbers during the most critical time of seed bank development. This suggests that under normal disturbance patterns these biological control agents will have little impact on the seed bank size and the long-term survival of seeds in the soil of their invasive Australian *Acacia* hosts [54].

This predicted lack of impact of seed reducing agents on the seed bank of invasive Australian *Acacia* is supported by the lack of evidence that the gall-forming biological control agent of *A*. *saligna* is having an impact on its seed banks. This is despite the 15 to 30 year presence of *U*. *tepperianum* within stands of *A*. *saligna* across South Africa. These findings support earlier observations by Strydom [57]. The lack of impact by *U*. *tepperianum* may further be ascribed to the fact that many of the seeds that are lost to this agent, in its absence would have been lost to natural processes such as seed abortion [58], pre-dispersal predation [59,60], post-dispersal predation [61], germination and decay [14].

The observation that many seeds survive and accumulate in the seed banks of invasive Australian *Acacia* despite the impact gall-forming agents has been recognised with the release of additional seed reducing agents i.e. the seed feeding weevils [15]. Most of the *Melanterius* species have been present in South Africa for too short a period of time to assess their impact. However, these seed reducing agents have similar deficiencies to the fungus, namely slow rates of dispersal and population growth relative to rate of seed bank accumulation of their Australian *Acacia* hosts [15,56]. This can be seen from the current accumulation of the seed bank of *A*. *longifolia* over time despite the presence of both its biological control agents (*Trichilogaster acaciaelongifoliae* and *Melanterius ventralis*) for over 30 years in South Africa.

The relationship between seed banks and the stem diameter of Australian *Acacia* can be used to manage invasive stands of these plants more effectively. Stands should not be cleared when seed bank input is at a maximum, as suggested by stem diameter measurements. Rather, management should be conducted when the potential seed bank size is the smallest (i.e. at low or high stem diameter). In practise this translates into targeting areas disturbed by fire, and climax stands of large trees.

Large uniform stands of senescent trees are rare as a consequence of continual patch generation [6,17]. Australian *Acacia* populations can change rapidly in terms of tree size and density over relatively short spatial scales (personal obs.). Consequently, managing seed banks underneath trees that have become senescent will require working on very small scales. Identifying these patches will also require constant fine scale measurements to be taken. Therefore, targeting old stands or patches with senescent trees for management may be impractical. Besides being impractical, leaving stands to senesce is also undesirable as these stands will still exert their negative impacts (e.g. increasing nutrients, decreasing water availability etc.) on the environment [2]. In addition to the negative impacts these stand exert over time, they will also serve as invasion foci from which these plants can spread [62].

Managing seed banks during the initial stages of stand development, after a fire or large scale mechanical clearing event, will be the most practical as stands (i.e. germinating seedlings) will be more uniformly distributed in terms of tree age and size. A prioritisation model developed by Roura-Pascaul et al. [63] also indicated that management should be conducted shortly after a fire event. It was concluded that seed reserves of invasive plants drastically decrease after such events and that the emerging seedlings could be removed before they mature. Managing seedlings rather than mature trees will be more cost-effective [63]. Targeting the early stages of Australian *Acacia* population development will also ultimately decrease the impact of these plants on the environment and decrease seed bank size and the long-term persistence of seeds in the soil. After initial clearing events, continual follow up clearing operations will be required to manage continued inter-fire recruitment [6,7]. If areas are kept clear from these trees the residual seed banks should decline as a consequence of germination and decay [7,14]. However, long term follow up clearing operations will be required for the seeds that are able to remain dormant for 50 or more years [6].

The results of this study do, however, run counter to current control actions which do not prioritise old stands, which are instead passively managed through biological control agents [16]. We have demonstrated negligible impact of seed-reducing biological control agents on seed bank accumulation of *A*. *longifolia*, *A*. *pycnantha* and *A*. *saligna*. This counters the conclusions of De Lange and Van Wilgen [64] which regard the benefit of seed biological control agents of Australian *Acacia* to far outweigh the cost of agent importation. We propose that mechanical clearing of seedlings (less than two years old) after disturbance as well as mechanical clearing of old stands to be a more effective management option for dealing with invasive Australian *Acacia*. This study thus provides the first evidence and recommendations for managing invasive Australian *Acacia* based on seed bank size development over time.

## Conclusion

Australian *Acacia* seed banks are still large over their introduced ranges in the Western Cape of South Africa, despite the long-term presence of gall-forming biological control agents. The seed bank data suggests that annual seed input is still high and that many seeds survive the pre-dispersal phase. Therefore, the current seed banks of the studied Australian *Acacia* are not residual (i.e. due to seed inputs before biological control agent release). There is no evidence that the current biological control agents are having an effect on the seed bank size of their targeted hosts. It is proposed that seed banks can be managed through using stem diameter to indicate potential seed bank size. Long term active management (mechanical), despite its higher cost to biological control, is required in order to manage the seed banks of invasive Australian *Acacia* effectively.

## Supporting information

S1 Fig Study site locations. Study sites over the sampled distribution range in the Western Cape of South Africa. AV—Avondvrede; DK—De Kijker; DL—De Liefde; FB—Fabel; FF—Fairfield; FG—Fraaigelegen; HB—Heuningbos; HV—Haasvlakte; IV—Iddasvallei; LH—Locheim; LM—Lio Marico; MP—Mooiplaas; MR—Modderrivier; MV—Meulvlakte; PN—Paarl; RD—Rivendale; SD—Squaredale; VV—Vaalvlei; WB—Waboomsrivier; WL—Wolseley; WR—White River. Three letters indicate locations where more than one species was present with the last letter indicating the species identity: L—*A*. *longifolia*, M—*A*. *mearnsii*, P—*A*. *pycnantha*, S—*A*. *saligna*.
(TIF)

S2 Fig Quantile regression analysis with extreme seed bank values for *Acacia longifolia*. A) The response of the seed bank (m^-2^) of *Acacia longifolia*, with the extreme values of sites included, to stem diameter, estimated by 95% quantile regressions. B) Average seed bank size m^-2^ (+SE) of *A*. *longifolia* according to stem diameter classes.
(TIFF)

S1 Table Long-term climatic parameters for study sites. Site co-ordinates, altitude (Alt), mean annual precipitation (mm), winter concentration of precipitation (WCP), mean annual temperature (MAT), average minimum temperature of the coldest month (Tn) and the average maximum temperature of the warmest month (Tx) of 26 ant-dispersed Australian *Acacia* study sites in the Western Cape of South Africa.
(DOCX)

S2 Table Invasive Australian *Acacia* seed bank size before and after biological control release in the Western Cape of South Africa. Seed bank estimates before and after the release of the respective biological control agents as determined by previous seed bank studies. The locality at which the samples were taken, the year of sampling, biological control agent presence including biological control agent identity is shown.
(DOCX)

S3 Table Regression line parameter estimates for studied Australian *Acacia*. Parameter estimates of the fitted response functions in Figure 2.2 (A) of the studied Australian *Acacia* species. Response functions were fitted through using quantile regression.
(DOCX)

S1 File *Acacia longifolia* extreme values. Extreme values were removed because of the effect of these data points on the regression analysis.
(DOCX)

S1 Dataset (XLSX)
