# Supplementary figures and images for: Invasive Australian Acacia seed banks: Size and relationship with stem diameter in the presence of gall-forming biological control agents
Source: PLoS One. 2017 Aug 16;12(8):e0181763. doi: 10.1371/journal.pone.0181763 (PMC5558976; doi:10.1371/journal.pone.0181763)

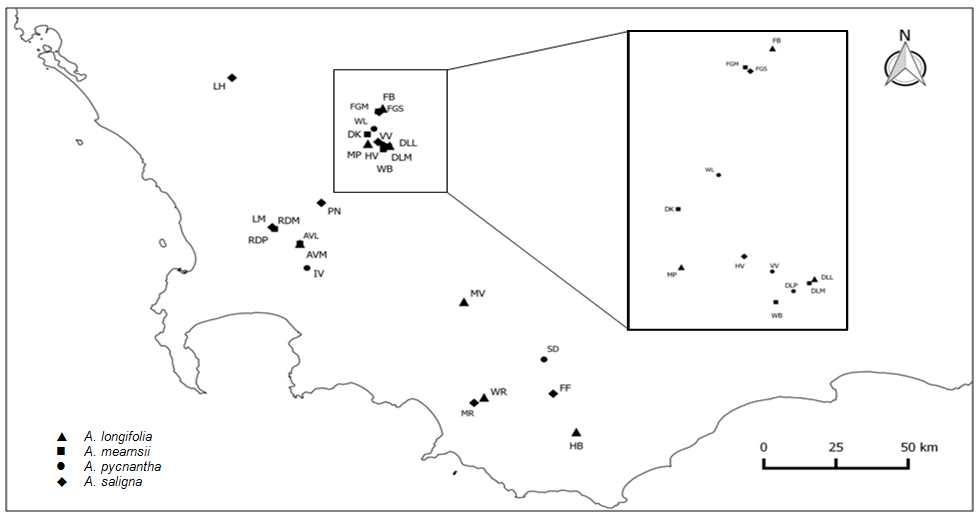

Supplement: S1 Fig — Study sites over the sampled distribution range in the Western Cape of South Africa. AV—Avondvrede; DK—De Kijker; DL—De Liefde; FB—Fabel; FF—Fairfield; FG—Fraaigelegen; HB—Heuningbos; HV—Haasvlakte; IV—Iddasvallei; LH—Locheim; LM—Lio Marico; MP—Mooiplaas; MR—Modderrivier; MV—Meulvlakte; PN—Paarl; RD—Rivendale; SD—Squaredale; VV—Vaalvlei; WB—Waboomsrivier; WL—Wolseley; WR—White River. Three letters indicate locations where more than one species was present with the last letter indicating the species identity: L—A. longifolia, M—A. mearnsii, P—A. pycnantha, S—A. saligna. (TIF) [file pone.0181763.s001.tif]

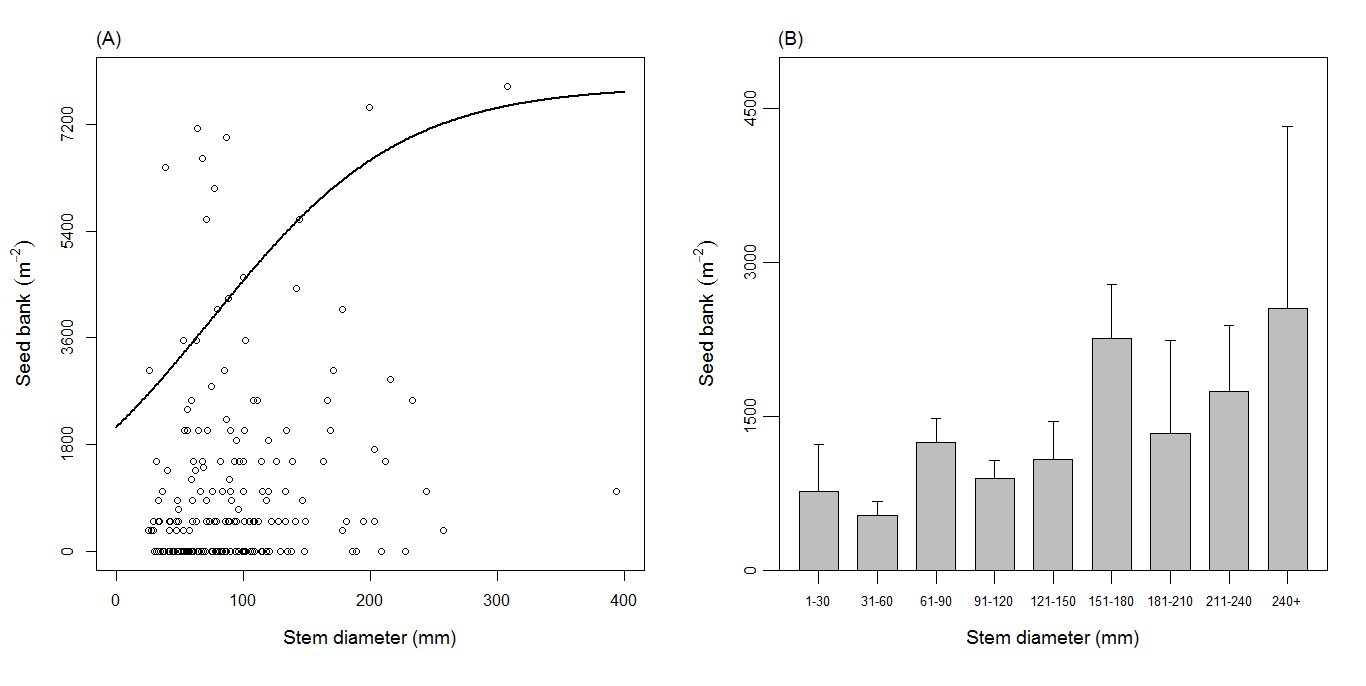

Supplement: S2 Fig — A) The response of the seed bank (m-2) of Acacia longifolia, with the extreme values of sites included, to stem diameter, estimated by 95% quantile regressions. B) Average seed bank size m-2 (+SE) of A. longifolia according to stem diameter classes. (TIFF) [file pone.0181763.s002.tiff]
