# Supplementary material for: Invasive Australian Acacia seed banks: Size and relationship with stem diameter in the presence of gall-forming biological control agents
Source: PLoS One. 2017 Aug 16;12(8):e0181763. doi: 10.1371/journal.pone.0181763 (PMC5558976; doi:10.1371/journal.pone.0181763)
Supplement: S1 Table — Site co-ordinates, altitude (Alt), mean annual precipitation (mm), winter concentration of precipitation (WCP), mean annual temperature (MAT), average minimum temperature of the coldest month (Tn) and the average maximum temperature of the warmest month (Tx) of 26 ant-dispersed Australian Acacia study sites in the Western Cape of South Africa. (DOCX) [file pone.0181763.s003.docx]

| **Site** | **Species** | **Co-ordinates** | **Alt (m)** | **MAP (mm)** | **WCP (%)** | **MAT (°C)** | **Tn (°C)** | **Tx (°C)** |
| --- | --- | --- | --- | --- | --- | --- | --- | --- |
| Fable | Al | S 33° 21’ E 19° 13’ | 418 | 598 | 80 | 18 | 6 | 32 |
| Mooiplaas | Al | S 33° 28’ E 19° 09’ | 276 | 598 | 80 | 18 | 6 | 32 |
| De liefde | Al | S 33° 29’ E 19° 14’ | 370 | 598 | 80 | 18 | 6 | 32 |
| Avondvrede | Al | S 33° 49’ E 18° 52’ | 223 | 689 | 75 | 18 | 9 | 31 |
| Meulvlakte | Al | S 33° 03’ E 19° 29’ | 237 | 456 | 66 | 17 | 7 | 29 |
| White river | Al | S 34° 24’ E 19° 32’ | 31 | 748 | 63 | 17 | 10 | 27 |
| Heuningbos | Al | S 34° 33’ E 19° 52’ | 149 | 452 | 57 | 17 | 6 | 27 |
| Fraaigelegen | Am | S 33° 21’ E 19° 12’ | 299 | 598 | 80 | 18 | 6 | 32 |
| De Kijker | Am | S 33° 26’ E 19° 09’ | 295 | 598 | 80 | 18 | 6 | 32 |
| De Liefde | Am | S 33° 29’ E 19° 14’ | 346 | 598 | 80 | 18 | 6 | 32 |
| Waboomsrivier | Am | S 33° 29’ E 19° 12’ | 263 | 598 | 80 | 18 | 6 | 32 |
| Rivendale | Am | S 33° 46’ E 18° 47’ | 181 | 576 | 75 | 18 | 8 | 30 |
| Avondvrede | Am | S 33° 49’ E 18° 52’ | 223 | 689 | 75 | 18 | 9 | 31 |
| Wolseley | Ap | S 33° 25’ E 19° 10’ | 272 | 598 | 80 | 18 | 6 | 32 |
| Vaalvlei | Ap | S 33° 28’ E 19° 12’ | 274 | 598 | 80 | 18 | 6 | 32 |
| De liefde | Ap | S 33° 29’ E 19° 13’ | 295 | 598 | 80 | 18 | 6 | 32 |
| Rivendale | Ap | S 33° 46’ E 18° 47’ | 178 | 576 | 75 | 17 | 8 | 30 |
| Iddasvalley | Ap | S 33° 55’ E 18° 54’ | 244 | 955 | 78 | 17 | 6 | 30 |
| Squaredale | Ap | S 33° 17’ E 19° 46’ | 250 | 439 | 60 | 15 | 6 | 27 |
| Locheim | As | S 33° 13’ E 18° 39’ | 149 | 416 | 81 | 19 | 8 | 33 |
| Fraaigelegen | As | S 33° 21’ E 19° 12’ | 309 | 598 | 80 | 18 | 6 | 32 |
| Haasvlakte | As | S 33° 28’ E 19° 11’ | 253 | 598 | 80 | 18 | 6 | 32 |
| Lio Marico | As | S 33° 45’ E 18° 46’ | 143 | 576 | 75 | 18 | 8 | 30 |
| Paarl | As | S 33° 41’ E 18° 58’ | 100 | 688 | 81 | 18 | 8 | 31 |
| Modderrivier | As | S 34° 25’ E 19° 30’ | 29 | 748 | 63 | 17 | 10 | 27 |
| Fairfield | As | S 33° 24’ E 19° 48’ | 141 | 412 | 60 | 17 | 5 | 29 |

**Table S1. Long term climatic parameters for study sites.**

Site co-ordinates, altitude (Alt), mean annual precipitation (mm), winter concentration of precipitation (WCP), mean annual temperature (MAT), average minimum temperature of the coldest month (Tn) and the average maximum temperature of the warmest month (Tx) of 26 ant-dispersed Australian *Acacia* study sites in the Western Cape of
South Africa.

Climatic data for the past 20 years were acquired from the ARC-ISCW. Weather stations within a 25 km radius from study sites and situated at approximately the same altitude were chosen. The altitude of sampling locations and weather stations were determined through the use of Google earth.
