# Supplementary material for: Invasive Australian Acacia seed banks: Size and relationship with stem diameter in the presence of gall-forming biological control agents
Source: PLoS One. 2017 Aug 16;12(8):e0181763. doi: 10.1371/journal.pone.0181763 (PMC5558976; doi:10.1371/journal.pone.0181763)
Supplement: S2 Table — Seed bank estimates before and after the release of the respective biological control agents as determined by previous seed bank studies. The locality at which the samples were taken, the year of sampling, biological control agent presence including biological control agent identity is shown. (DOCX) [file pone.0181763.s004.docx]

| **Species** | **Site** | **Year** | **Seed bank m^-2^** | **Sample size** | **Biocontrol before/after** | **Agent identity** | **References** |
| --- | --- | --- | --- | --- | --- | --- | --- |
| *A. cylops* | Rondevlei | 1981 | 1571 | 50 | before | na | Milton & Hall 1981 |
|  | Rondevlei | 1985 | 1400 | 60 | before | na | Holmes et al. 1987 |
|  | Rondevlei | 1986/1987 | 2060 | 50 | before | na | Holmes & Rebelo 1988 |
|  | Goukamma Nature reserve | 1985 | 1370 | 179 | before | na | Holmes et al. 1987 |
|  | Goukamma | 1986/1987 | 3720 | 50 | before | na | Holmes & Rebelo 1988 |
|  | Buffels Bay | 1985 | 3650 | 60 | before | na | Holmes et al. 1987 |
|  | Walker Bay Forest Reserve | 1985 | 5140 | 219 | before | na | Holmes et al. 1987 |
|  | Walker Bay Forest Reserve | 1985 | 2832-7792 | 50 | before | na | Holmes 1989b |
|  | Walker Bay | 1986/1987 | 4100 | 50 | before | na | Holmes & Rebelo 1988 |
|  | Klipfontein | 1986/1987 | 1590 | 50 | before | na | Holmes & Rebelo 1988 |
|  | Fairvlei | 1986/1987 | 2670 | 50 | before | na | Holmes & Rebelo 1988 |
|  | Koeberg | 1986/1987 | 3450 | 50 | before | na | Holmes & Rebelo 1988 |
|  | Homestead | 1986/1987 | 7930 | 50 | before | na | Holmes & Rebelo 1988 |
|  | Renosterkop | 1986/1987 | 4480 | 50 | before | na | Holmes & Rebelo 1988 |
|  | Arniston | 1986/1987 | 11400 | 50 | before | na | Holmes & Rebelo 1988 |
|  | Worcester | 1986/1987 | 2510 | 50 | before | na | Holmes & Rebelo 1988 |
|  | Albertina | 1986/1987 | 1290 | 50 | before | na | Holmes & Rebelo 1988 |
|  | Keurboom | 1986/1987 | 2370 | 50 | before | na | Holmes & Rebelo 1988 |
|  | St. Francis | 1986/1987 | 3700 | 50 | before | na | Holmes & Rebelo 1988 |
|  | Port Elizabeth | 1986/1987 | 5680 | 50 | before | na | Holmes & Rebelo 1988 |
|  | Port Alfred | 1986/1987 | 1380 | 50 | before | na | Holmes & Rebelo 1988 |
|  | East London | 1986/1987 | 28 | 50 | before | na | Holmes & Rebelo 1988 |
| *A. longifolia* | University of Cape Town | 1981 | 2110 | 50 | before | na | Milton & Hall 1981 |
|  | Banhoek Valley | 1982 | 2078-2901 | 30 | after | TA | Pieterse & Cairns 1986 |

**Table S2. Invasive Australian *Acacia* seed bank size before and after biological control release in the Western Cape of South Africa.**

Seed bank estimates before and after the release of the respective biological control agents as determined by previous seed bank studies. The locality at which the samples were taken, the year of sampling, biological control agent presence including biological control agent identity is shown.

na: indicates not applicable. UT: *Uromycladium tepperianum*. MC: *Melanterius compactus*. TA: *Trichilogaster acaciaelongifoliae*

**Table S2. Continued**

| **Species** | **Site** | **Year** | **Seed bank m^-2^** | **Sample size** | **Biocontrol before/after** | **Agent identity** | **References** |
| --- | --- | --- | --- | --- | --- | --- | --- |
| *A. saligna* | Penhill | 1981 | 11070 | 50 | before | na | Milton & Hall 1981 |
|  | Faure | 1981 | 7586 | 50 | before | na | Milton &Hall 1981 |
|  | Marina da Gama | 1981 | 145 | 50 | before | na | Milton & Hall 1981 |
|  | Grootphisantekraal | 1985 | 45800 | 50 | before | na | Holmes et al. 1987 |
|  | Buffels Bay | 1985 | 13200 | 60 | before | na | Holmes et al. 1987 |
|  | Cape Flats Nature Reserve | 1985 | 22400 | 60 | before | na | Holmes et al. 1987 |
|  | Coppul | 1991 | 2000 | 40 | after | UT | Morris 1997 |
|  | Swartwater | 1991 | 3000 | 40 | after | UT | Morris 1997 |
|  | Kanonkop | 1991 | 4000 | 40 | after | UT | Morris 1997 |
|  | Riverlands | 1991 | 7000 | 40 | after | UT | Morris 1997 |
|  | Hutch’s Place | 1991 | 21000 | 40 | after | UT | Morris 1997 |
|  | Burgerspos | 1991 | 22000 | 40 | after | UT | Morris 1997 |
|  | Sunvalley | 2003 | 13295 | 30 | after | UT and MC | Jason 2005 |
|  | Noorhoek | 2003 | 46355 | 30 | after | UT and MC | Jason 2005 |
|  | Steenberg | 2003 | 3888 | 30 | after | UT and MC | Jason 2005 |
|  | Simonstown | 2004 | 3158 | 30 | after | UT and MC | Jason 2005 |
|  | Burgespos | 2009 | 24452 | 30 | after | UT and MC | Strydom et al. 2012 |
|  | Swartwater | 2009 | 20468 | 30 | after | UT and MC | Strydom et al. 2012 |
|  | Travellers Rest | 2010/2011 | 8483 - 37316 | 50 | after | UT and MC | Strydom 2012 |
|  | Citrusdal | 2010/2011 | 12865 - 28996 | 50 | after | UT and MC | Strydom 2012 |
|  | Rietvlei | 2010/2011 | 2254 - 6806 | 50 | after | UT and MC | Strydom 2012 |
|  | Soutvlakte | 2010/2011 | 6459 - 12648 | 50 | after | UT and MC | Strydom 2012 |
|  | Veldrift | 2010/2011 | 4726 - 11738 | 50 | after | UT and MC | Strydom 2012 |
|  | Bossiesvlei | 2010/2011 | 27021 - 33342 | 50 | after | UT and MC | Strydom 2012 |
|  | Locheim | 2010/2011 | 13296 - 24620 | 50 | after | UT and MC | Strydom 2012 |
|  | Swartwater | 2010/2011 | 19368 - 21086 | 50 | after | UT and MC | Strydom 2012 |

na: indicates not applicable. UT: *Uromycladium tepperianum*. MC: *Melanterius compactus*. TA: *Trichilogaster acaciaelongifoliae*

**Table S2. Continued**

| **Species** | **Site** | **Year** | **Seed bank m^-2^** | **Sample size** | **Biocontrol before/after** | **Agent identity** | **References** |
| --- | --- | --- | --- | --- | --- | --- | --- |
| *A. saligna* | Yzerfontein | 2010/2011 | 3042 - 7395 | 50 | after | UT and MC | Strydom 2012 |
|  | Romansrivier | 2010/2011 | 2610 - 6186 | 50 | after | UT and MC | Strydom 2012 |
|  | Burgerspost | 2010/2011 | 21590 - 22541 | 50 | after | UT and MC | Strydom 2012 |
|  | Kanonkop | 2010/2011 | 1500 - 2174 | 50 | after | UT and MC | Strydom 2012 |
|  | Goudiniweg | 2010/2011 | 7932 - 14127 | 50 | after | UT and MC | Strydom 2012 |
|  | Positano | 2010/2011 | 16866 - 23931 | 50 | after | UT and MC | Strydom 2012 |
|  | Vergenoegd | 2010/2011 | 968 - 1334 | 50 | after | UT and MC | Strydom 2012 |
|  | Hutch’s Place | 2010/2011 | 3881 - 6570 | 50 | after | UT and MC | Strydom 2012 |
|  | Buffelsrivier | 2010/2011 | 137 - 1040 | 50 | after | UT and MC | Strydom 2012 |
|  | Rooisand | 2010/2011 | 2394 - 4919 | 50 | after | UT and MC | Strydom 2012 |
|  | Coppul | 2010/2011 | 888 - 2200 | 50 | after | UT and MC | Strydom 2012 |
|  | Modderrivier | 2010/2011 | 910 - 2815 | 50 | after | UT and MC | Strydom 2012 |
|  | Fairfield | 2010/2011 | 2045 - 5941 | 50 | after | UT and MC | Strydom 2012 |
|  | Haasvlakte | 2010/2011 | 9805 - 14505 | 50 | after | UT and MC | Strydom 2012 |
|  | Môreson | 2010/2011 | 6207 - 9334 | 50 | after | UT and MC | Strydom 2012 |
|  | Kragga Kamma | 2010/2011 | 6752 - 7161 | 50 | after | UT and MC | Strydom 2012 |
|  | Welgelegen | 2010/2011 | 3799 - 6264 | 50 | after | UT and MC | Strydom 2012 |

na: indicates not applicable. UT: *Uromycladium tepperianum*. MC: *Melanterius compactus*. TA: *Trichilogaster acaciaelongifoliae*
