# Supplementary material for: Invasive Australian Acacia seed banks: Size and relationship with stem diameter in the presence of gall-forming biological control agents
Source: PLoS One. 2017 Aug 16;12(8):e0181763. doi: 10.1371/journal.pone.0181763 (PMC5558976; doi:10.1371/journal.pone.0181763)
Supplement: S3 Table — Parameter estimates of the fitted response functions in Figure 2.2 (A) of the studied Australian Acacia species. Response functions were fitted through using quantile regression. (DOCX) [file pone.0181763.s005.docx]

**Table S3. Regression line parameter estimates for studied Australian *Acacia.***

| **Species** | **a** | **b** | **c** | **d** |
| --- | --- | --- | --- | --- |
| *A. pycnantha* | -2.10266717 | 0.01207437 | 5.50503284 | 0.13091288 |
| *A. saligna* | -2.36188869 | 0.02837727 | 3.75778983 | 0.10072261 |
| *A. mearnsii* | -1.61892343 | 0.01289262 | 3.39226432 | 0.05814434 |
| *A. longifolia* | -3.15540063 | 0.01185913 | 1.92953851 | 0.03227207 |

Parameter estimates of the fitted response functions in Figure 2.2 (A) of the studied Australian *Acacia* species. Response functions were fitted through using quantile regression
