# Supplementary material for: Invasive Australian Acacia seed banks: Size and relationship with stem diameter in the presence of gall-forming biological control agents
Source: PLoS One. 2017 Aug 16;12(8):e0181763. doi: 10.1371/journal.pone.0181763 (PMC5558976; doi:10.1371/journal.pone.0181763)
Supplement: S1 File — Extreme values were removed because of the effect of these data points on the regression analysis. (DOCX) [file pone.0181763.s006.docx]

#### Acacia longifolia extreme values

The extreme values of *A. longifolia* (>6000 seeds m^-2^) were removed. These points were removed, as exclusion of one of these points, at higher stem diameter, changed the relationship between the seed bank and stem diameter. Schröder et al. (2005) also state in their supplementary material that the nlrq-package in R often fails to fit extreme quantiles in the usually small data sets of vegetation science.
